# Health benefits conferred by the human gut microbiota during infancy

**DOI:** 10.1111/1751-7915.13334

**Published:** 2018-11-08

**Authors:** Marco Ventura, Christian Milani, Gabriele A. Lugli, Douwe van Sinderen

**Affiliations:** ^1^ Laboratory of Probiogenomics Department of Chemical Sciences, Life Sciences and Environmental Sustainability University of Parma Parma Italy; ^2^ APC Microbiome Institute and School of Microbiology National University of Ireland Cork Ireland

## Abstract

Development of the human gut throughout the entire life.

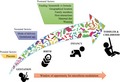

## General features

The human gut microbiota represents an extraordinary complex microbial consortium whose activities are believed to be crucial for human health and well‐being. The formation of the human gut microbiota commences at the point of delivery of an essentially sterile baby and then further develops in terms of diversity and complexity during weaning, adolescence and into adult life. The microbial groups identified in the gut microbiota encompass representatives of all three domains of life, i.e. Archaea, Bacteria and Eukarya, whose dynamics are manoeuvred by various forces, such as viruses, which are believed to act as powerful drivers of gut microbiota homoeostasis (for a review see Ref.: Milani *et al*., 2017). The human gut microbiota is composed of autochthonous members that are believed to establish stable interactions with their host, as well as a transient microbial consortium whose members are mainly acquired through oral intake and who are not presumed to colonize the gut in the presence of a homoeostatic microbiota. The diverse components of the gut microbiota are known to elicit key effects in the maintenance and promotion of human health by (i) supporting the transformation of food components so as to release nutrients that would otherwise not be accessible to the host, (ii) stimulating host cell differentiation, (iii) defending the body against pathogen invasion and (iv) exerting immune modulatory activities. Several epidemiological studies have clearly demonstrated a correlation between factors disrupting the gut microbiota during childhood and the occurrence of certain immune and metabolic disorders at later stages of life (Eggesbo *et al*., 2003; Huh *et al*., 2012; Sevelsted *et al*., 2015), backed up by other scientific data that link long‐term health benefits to the infant gut microbiota (Relman, 2012). This scientific realization has in turn opened up the search for strategies that aim to influence the origin, development, maturation and activities of the infant microbiota by a plethora of nutraceutical/functional food products including probiotics and prebiotics.

An interesting feature of the human gut microbiota is that the development of such a microbial assemblage reaches homoeostasis, which is characterized by a compositional equilibrium of its microbial members (Tamboli *et al*., 2004). Various events may cause disturbances in this so‐called microbiota climax, thereby causing a state that has been referred to as dysbiosis. The concept of dysbiosis is controversial, simply because there is still a lack of knowledge and understanding of what precisely represents a ‘normal’ or healthy microbiota. Dysbiosis is usually associated with harmful effects and may have long‐term consequences leading to disorders or diseases, including obesity, diabetes and inflammatory bowel disease (IBD). However, one should realize that significant variations occur in gut microbiota composition during host development from infancy to early childhood, from young to ageing adults and during pregnancy. Even if such modifications appear to be similar to those described in certain disease conditions, in their evolving context they do not necessarily lead to illness but, rather, may enhance fitness and survival and thus are considered to be beneficial (Nuriel‐Ohayon *et al*., 2016).

## The origin of the human gut microbiota

**Figure 1:**
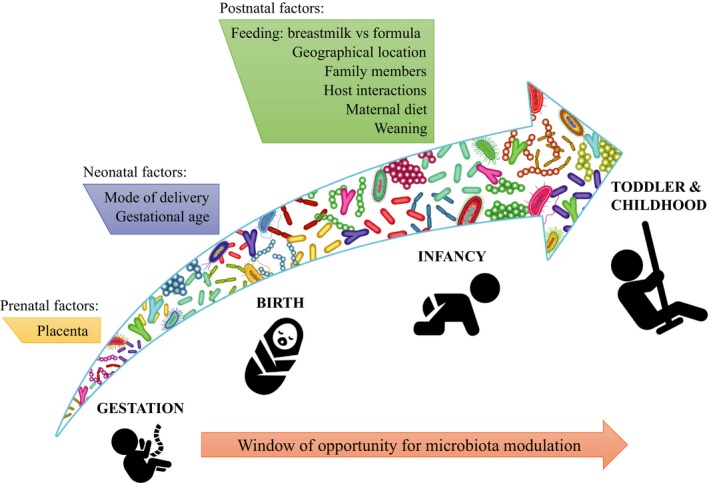
Development of the human gut throughout the entire life. The diagram describes a list of prenatal, neonatal and postnatal influences that may contribute to the establishment/maturation/succession of bacterial consortia in the human gut.

The establishment of the infant gut microbiota is the result of a complex process that is influenced by many environmental and host factors (Rodriguez *et al*., 2015) (Fig. 1). Notably, although the dogma of a sterile *in utero* environment, which claims that microbial colonization only commences upon birth, is still generally accepted, there is growing evidence indicating that very early colonization of humans may occur at the fetal stage (Jimenez *et al*., 2005; DiGiulio *et al*., 2008; Aagaard *et al*., 2014). The route by which bacteria achieve this prebirth colonization is still obscure. However, the recent discovery of a circulating microbiota that is present in human blood (Kowarsky *et al*., 2017) is not only reinforcing the notion that there is not any human organ that is completely sterile, but also lending further support to the hypothesis of a prenatal establishment of the gut microbiota. This perception may facilitate new research avenues and possible manipulation of the human gut microbiota through probiotic intervention during pregnancy.

The progress and evolution of the gut microbiota is a dynamic and non‐casual process, involving intricate interactions between key microbial components (Avershina *et al*., 2013, 2016). This process is affected by several perinatal conditions, such as mode of delivery, type of feeding and antibiotic usage (Milani *et al*., 2017). In addition, diet, mother's age and metabolic status, family genetics and lifestyle have been described to influence the infant gut microbiota composition (Milani *et al*., 2017).

The origin of the human gut microbiota is associated with the inheritance of an assemblage of microorganisms that are vertically transmitted from mother to her offspring. Remarkably, the mammalian newborn does not appear to passively acquire a new microbiota, but the mother seems to actively promote the transfer of specific members of her microbiota to her offspring and may thus directly influence the development of the new microbiota of her child. This selective vertical transmission of microbes is driven by the specific nourishment with prebiotic milk compounds favouring certain microorganisms, in particular bifidobacteria. Recently, metagenomic investigations of mothers and corresponding newborns have highlighted the existence of shared bifidobacterial communities, in particular bifidobacterial strains that are capable of metabolizing specific glycans contained in human milk, i.e. human milk oligosaccharides (HMOs; Milani *et al*., 2015; Duranti *et al*., 2017). This vertical transmission route involving bifidobacterial strains includes human milk as a vectoring system (Milani *et al*., 2015; Duranti *et al*., 2017). A similar scenario seems to occur in other non‐human primates as well as in non‐primate mammals, where bifidobacterial strains were found to be maternally inherited by means of mother's milk (Milani *et al*., 2017). These findings support the hypothesis that those bifidobacteria that are directly acquired from mothers are maintained in the adult gut, perhaps at very low levels, in order to be ultimately transferred to the next generation (Milani *et al*., 2015). This intriguing process may therefore reflect millennia of co‐evolution between bifidobacteria and their mammalian host.

As mentioned above, human milk represents an important selective medium for the establishment and modulation of the gut microbiota during the early stages of life. In fact, human milk, apart from representing a source of nutrients for the infant, encompasses a large amount of carbohydrates such as HMOs; i.e., 1 l of human milk contains 5–20 g of these complex glycans, which is typically more than all human milk proteins combined and which acts as a highly selective nourishment for specific members of the infant gut microbiota. Notably, HMOs encompass a diverse group of complex glycans, where five monosaccharide building blocks glucose, galactose, N‐acetylglucosamine, fucose and N‐acetyl‐neuraminic acid are arranged in a variety of combinations, which generates more than 200 structurally distinct HMOs (Bode, 2012; Milani *et al*., 2017). Furthermore, it should be noted that HMO content and composition depend on lactation stage, the genetics of the mother and environmental factors (McGuire *et al*., 2017). Therefore, human milk can be considered as a ‘personalized, mother‐specific nutrition’ and HMOs as ‘personalized prebiotics’, together promoting the arrangement of a distinct infant gut microbiome, the composition of which being determined, at least in part, by the HMOs present in mother's milk. Every mother provides a different HMO mix to her infant(s), determining distinct infant microbial assemblies with potential short‐ and long‐term effects (Milani *et al*., 2017).

Only a small number of bacteria, such as the *Bifidobacterium longum* subsp. *infantis* ATCC 15697 and *Bifidobacterium bifidum* PRL2010, have been shown to possess the enzymatic machinery to metabolize many if not all HMOs present in human milk (Sela *et al*., 2008; Turroni *et al*., 2010). In contrast, other bacteria can access and metabolize only specific elements of the HMO mixture. Nevertheless, microbial communities may be capable to act in concert, consecutively breakdown and metabolize HMO structures in a team effort through cross‐feeding activities (Egan *et al*., 2014a,b; Gotoh *et al*., 2018).

## The early microbiota as a key determinant of human health

In the course of the initial microbial colonization, the gut microbiota is still unstable and is going through continuous modifications until the infant is 2–3 years old, at which stage the microbiota reaches a composition resembling that of an adult (Yatsunenko *et al*., 2012). Despite this apparent instability of the infant gut microbiota, it is very well known that microbiota–host communications are particularly important during these early stages of life. In fact, during this period, microbiota–host interactions represent key events underpinning immune development of the human host, with consequent creation and conservation of the homoeostasis of the host microbiota during infancy. Remarkably, these actions have not only immediate health effects but also long‐term health consequences. In this context, the infant gut microbiota provides a strong antigenic stimulus needed for the maturation of the gut and associated immune functions (Sommer and Backhed, 2013; Gensollen *et al*., 2016). This effect also impacts on the development of the distal organs, by influencing the host at systemic level (Clarke *et al*., 2014; Neuman *et al*., 2015). Consequently, it is accepted that the foundation of human health is linked with the establishment and development of the microbiota (Arrieta *et al*., 2014; Nylund *et al*., 2014). In this context, it has been shown that faecal levels of IgA, which is considered to be an important marker linked to the risk of disease, may be associated with the presence of specific microbiota members (Moon *et al*., 2015). Thus, it may be argued that any modification from the normal neonatal microbial colonization process represents a risk factor for disease later in life. Certainly, accumulating scientific evidence indicates that gut microbiota alterations during the first stages of life are predictors of disease development (Kalliomaki *et al*., 2008; Fujimura *et al*., 2016; Simonyte Sjodin *et al*., 2016). In this context, it should also be noted that the early microbiota is connected to infant malnutrition and associated growth impairment (Arboleya *et al*., 2012; Gough *et al*., 2015; Reyes *et al*., 2015; Charbonneau *et al*., 2016).

There is a long list of studies involving immune pathologies such as atopic eczema and asthma, IBD, irritable bowel syndrome (IBS), type 1 diabetes (T1D), metabolic disorders, like obesity type 2 diabetes, displaying a clear correlation with alteration in the early gut microbiota.

## The infant gut microbiota and its impact on the host health

An incorrect establishment of the gut microbiota during the early stages of life has been shown to be associated to disruption of immune homoeostasis, ultimately impacting on the risk of allergic diseases later in life (Johnson and Ownby, 2016). Interestingly, microbiota differences related to low loads of certain bacterial taxa such as bifidobacteria and other intestinal anaerobes such as *Faecalibacterium prausnitzii* and *Coprococcus eutactus* were identified in infants that later developed atopy or asthma (Fujimura *et al*., 2016). Even if these results do not connect an early microbiota profile with a decrease of atopic eczema disorder, they do indirectly highlight the protective role of a butyrate‐producing microbiota against the development of atopic eczema. In fact, it has been shown that a low relative abundance of butyrate producers is a risk predictor for the development of atopic eczema (Simonyte Sjodin *et al*., 2016).

Several studies have reported a clear association between early live antibiotic exposure and childhood obesity (Cox and Blaser, 2015). These findings indicate that antibiotics‐mediated disturbances of the gut microbiota, either during prenatal or postnatal periods, enhance the risk of obesity (Turta and Rautava, 2016). Recently, it has been proposed that early microbial profiles may predict overweight in children. In this context, it has been shown that the bifidobacterial loads at 6 and 12 months inversely correlate with overweight in children (Kalliomaki *et al*., 2008).

It has been proposed that microbiota alterations during early infancy cause a pro‐inflammatory environment that facilitates the occurrence of autoimmune disease such as T1D (Kostic *et al*., 2015). In particular, such microbiota perturbations include a lower diversity and significant differences in the ratio of the most abundant intestinal phyla, i.e. Firmicutes and Bacteroidetes, as well as a reduced load of the butyrate producer *F. prausnitzii* (Gulden *et al*., 2015). Notably, butyrate‐producing species have been suggested to exert a protective role in reducing the risk of developing T1D (de Goffau *et al*., 2014).

Several severe diseases involving intestinal inflammatory symptoms have been linked to deviations of the early life gut microbiota. The concept that the human gut immune system is educated by the early microbial colonization assumes a connection between pioneering microbial inhabitants of the human intestine and subsequent gut inflammatory disease/disorder, such as IBD and IBS. Interestingly, a number of clinical trials have indicated that early changes in the population of specific microbial groups herald the onset of intestinal inflammatory diseases at later stages of life (O'Mahony *et al*., 2009, 2014; Schirbel and Fiocchi, 2011; Wang *et al*., 2016). Nevertheless, conclusive data about the long‐lasting effects of dysbiosis in the early life and the development of IBD and IBS in later stages of life are rare and do not allow the establishment of causality.

## Conclusions

We are only beginning to unveil early life interactions between microbes and their mammalian host, and, if disturbed, the resulting long‐lasting effects on human health. Consequently, it may be time to develop alternative strategies to facilitate the appropriate establishment of the infant gut microbiota in those situations in which this process is prevented, challenged or disrupted.

This perception has fuelled the development of functional food strategies involving various nutraceutical products such as probiotics and/or prebiotics capable of modulating the infant gut microbiota establishment/maturation. However, such intervention approaches require thorough assessment of the composition of the human early gut microbiota and the identification of key microbiota components that play a role in the aetiology of diseases and that can thus act as microbial biomarkers. The identification of microbial biomarkers therefore represents an important activity for future prophylactic approaches as well as for early diagnosis of diseases.

We are only starting to understand the importance of microbiota composition during pregnancy in terms of a reservoir of bacteria for the establishment of gut microbiota in newborns. However, there are many open questions about how certain bacteria are specifically transferred by the mother to their children. Furthermore, we still do not know whether bacteria may transfer from mother to child through a systemic route, e.g. through dendritic cells, blood or milk. The answers to all these questions combined with the possibility of prenatal bacterial colonization may revolutionize paediatrics in the coming years.

## Conflict of interest

None declared.
